# Intra-Articular Injection of Adipose-Derived Stem Cells Ameliorates Pain and Cartilage Anabolism/Catabolism in Osteoarthritis: Preclinical and Clinical Evidences

**DOI:** 10.3389/fphar.2022.854025

**Published:** 2022-03-21

**Authors:** Bo Yan, Shuaijie Lv, Peijian Tong, Li Yan, Zuxiang Chen, Li Zhou, Qiang Yuan, Le Guo, Letian Shan

**Affiliations:** ^1^ College of Pharmaceutical Sciences, Zhejiang Chinese Medical University, Hangzhou, China; ^2^ The First Affiliated Hospital, Zhejiang Chinese Medical University, Hangzhou, China; ^3^ Cell Resource Bank and Integrated Cell Preparation Center of Xiaoshan District, Hangzhou Regional Cell Preparation Center (Shangyu Biotechnology Co., Ltd), Hangzhou, China

**Keywords:** adipose tissue-derived stem cells, osteoarthritis, paracrine, conditioned medium, treatment

## Abstract

**Background:** Osteoarthritis (OA) is the most common joint disorder, lacking disease-modifying treatments. Adipose-derived mesenchymal stem cells (ADSCs) are adult multipotent stromal cells obtained from fat tissue, which holds great potential in treating OA. This study aimed to evaluate the anti-OA efficacy of ADSCs from preclinical and clinical facets and explore the underlying mechanism of action.

**Methods:** *In vivo*, a single dose of 5 × 10^5^ ADSCs was injected into the knee joints of monoiodoacetate-induced OA rat model. The levels of metabolic and hypertrophic molecules (MMP13, Collagen II, Collagen X) of chondrocytes were measured by immunohistochemistry. *In vitro*, cell viability assay was conducted to detect the proliferation ability of chondrocytes treated with ADSCs conditioned medium (ADSCs-CM). Quantitative real-time polymerase chain reaction and Western blot assays were applied to explore the mechanism of action of ADSCs. Moreover, a retrospective analysis was conducted to determine the clinical efficacy and safety of ADSCs on OA patients.

**Results:** The animal study showed that ADSCs significantly alleviated OA cartilage lesions in rats, as was confirmed by downregulation of the MMP13 and Collagen X and upregulation of the Collagen II. *In vitro* data showed that ADSCs-CM promoted the proliferation of chondrocytes, and significantly restored the IL-1β-induced abnormal expressions of molecular markers IL-6, Aggrecan, MMP3, MMP13, Collagen II, Collagen X, ADAMTS5, ADAMTS9, SOX6, and SOX9 in chondrocytes. Such regulatory effects of ADSCs-CM on the proliferation and these anabolic, catabolic, and hypertrophic markers of chondrocytes suggested a paracrine-based mode of action of ADSCs. Furthermore, the clinical data showed that ADSCs reduced pain and repaired cartilage damage in OA patients, with no adverse events.

**Conclusion:** This study demonstrated the anti-OA efficacy, safety, and a paracrine-based mechanism of ADSCs, providing a promising cell-based therapeutic option for OA treatment.

## Introduction

Osteoarthritis (OA) is a degenerating joint disease characterized by progressive articular cartilage loss, subchondral bone sclerosis and synovial inflammation (Kulkarni et al., 2021; Sharma, 2021). It is the most common type of arthritis, affecting more than 500 million people worldwide, and accounting for 2% of total global years lived with disability (Hunter et al., 2020; Kulkarni et al., 2021). The OA incidence is expected to continuously increase due to the steady rise in obesity and ageing population (Hunter et al., 2020). As a leading cause of disability, OA significantly decreases the quality of life due to chronic pain, restricted mobility and psychological distress (Lentz et al., 2020; Toyoda et al., 2021). In addition to the physical and mental sufferings of OA patients, OA also brings a substantial economic burden to health care and society (Cross et al., 2020). Conventional treatments for low-degrade OA range from non-pharmacological approaches (reducing weight, physiotherapy, lifestyle changing) to pharmacological medications (nonsteroidal anti-inflammatory drugs, analgesics, corticosteroid, hyaluronic acid), which are helpful for symptomatic alleviation (Arden et al., 2021; Katz et al., 2021). However, none of these treatments seem to delay or reverse the progression of cartilage degeneration or have long-term improvement (Grässel and Muschter, 2020). For terminal-stage OA, joint arthroplasty or total replacement is currently the final option to recover joint movement, while it is often accompanied by infection-related complications and a second surgery is necessitated for early-onset OA patients (Billig et al., 2020; Chung et al., 2021). Therefore, new alternative therapies with better efficacy and safety are urgently needed.

The rapid development of cell-based therapies, such as autologous chondrocyte implantation (ACI) and mesenchymal stem cells (MSCs) injection, have greatly extended the choices of the regenerative medicine for cartilage repair (Fuggle et al., 2020). ACI has been successfully used to treat osteochondral defects to prevent the progression to OA (Gobbi et al., 2020b). Although initial clinical trials of ACI have shown modest results, the risks of two surgical procedures (harvest and implantation) and donor-site morbidity have greatly limited its wide clinical application (Gobbi et al., 2020a). Local inflammatory microenvironment of osteoarthritic joint also leads to low cell viability and poor synthesis of extracellular matrix (ECM) (Zheng et al., 2021). MSCs are adult multipotent stromal cells of mesodermal origin, which reside in diverse tissues, such as bone marrow, adipose tissue and umbilical cord (da Silva Meirelles et al., 2006). The high *in vitro* proliferation rate, chondrogenic differentiation potential, and immunomodulatory capabilities of MSCs are favored features for cartilage regeneration (Gupta et al., 2012). Besides, various anti-inflammatory molecules and growth-factors can be released from MSCs, which can create a suitable microenvironment for the survival of chondrocytes (da Silva Meirelles et al., 2009). Recent studies have demonstrated the beneficial effects of MSCs in treating OA (Wang et al., 2020; Maheshwer et al., 2021). Compared to the commonly used bone marrow-derived MSCs (BMSCs), adipose tissue-derived MSCs (ADSCs) are relatively easy to isolate *via* simple liposuction procedure and the cell yield is approximately 500 times higher than that of BMSCs from bone marrow aspiration (Rodriguez et al., 2005). Animal studies have shown that ADSCs could promote the expression of chondrocyte redifferentiation markers, thereby retarding the progression of OA (Zhang et al., 2021). Nonetheless, there is still some inconsistency between the results of preclinical and clinical studies using ADSCs for OA treatment, which requires further investigation (Agarwal et al., 2021).

The purpose of this study was to evaluate the anti-OA efficacy of ADSCs on monoiodoacetate (MIA)-induced rat OA model through pain behavior test, cartilage histopathological and immunohistochemical analyses. Considering the secreted soluble molecules from ADSCs, paracrine effect is expected to play a role in the regenerative process. Thus, ADSCs-conditioned medium (ADSCs-CM) was evaluated for the paracrine molecular mode of ADSCs. Moreover, a retrospective study was carried out to confirm the clinical efficacy of ADSCs on OA patients. The novelty of this study lies in the combination of animal experiment and clinical data to study the therapeutic activity of ADSCs, the evaluation of regulatory roles of ADSCs towards pain and cartilage metabolism through an animal model of OA pain, along with the determination of the paracrine action of ADSCs, demonstrating a promising anti-OA efficacy of ADSCs in clinic.

## Materials and Methods

### Reagents

Collagenase type IV and minimum essential medium-alpha modification (α-MEM) with Glutamax™-1 were purchased from Gibco (NY, United States). Fetal bovine serum (FBS) was purchased from CellMax (Beijing, China). IMDM (Iscove’s modified Dulbecco’s medium) and trypsin (0.25%) were purchased from Thermo Fisher Scientific (MA, United States). Monoiodoacetate (MIA) was purchased from Sigma-Aldrich. TRIzol reagent was purchased from TaKaRa Biotechnology Co. Ltd. (Dalian, China). All-in-One cDNA Synthesis SuperMix kit was purchased from Biotool (TX, United States). 2 × SYBR Green qPCR Master Mix (low ROX) kit was obtained from Bimake (TX, United States).

### Animals

**FIGURE 1:**
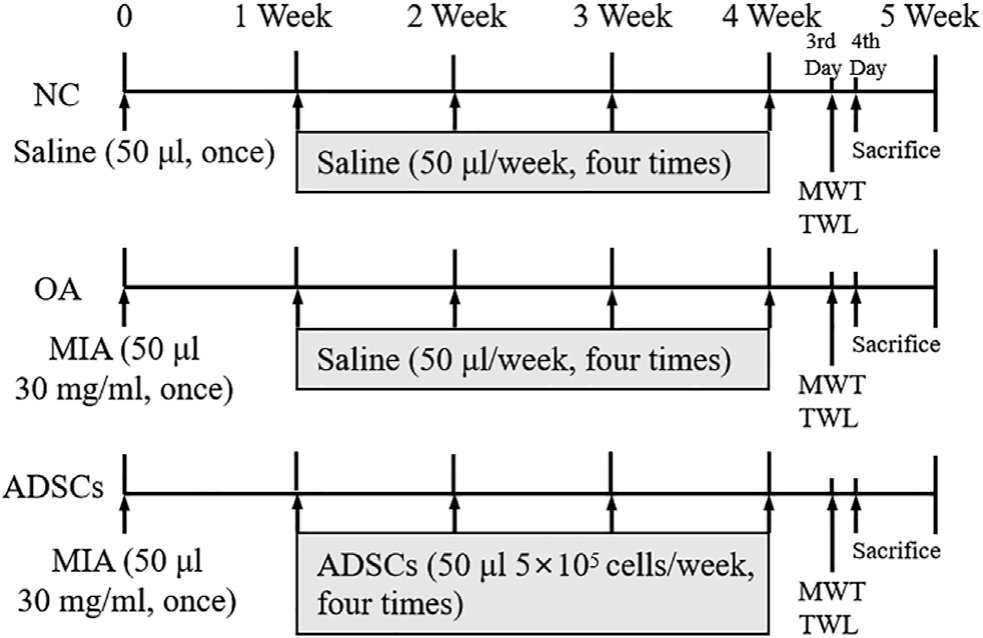
Timetable for the experimental design. OA model was generated with the intra-articularly injection of monoiodoacetate (MIA) in rats (7 weeks old) once and left for recovery for 1 week, followed by intra-articularly injection of saline or ADSCs for four consecutive weeks at the first day of the second, third, fourth and fifth week. NC, normal control group; OA, OA model group; ADSCs, ADSCs treated model group. At the third day of the fifth week, the mechanical withdrawal threshold (MWT) and the thermal withdrawal latency (TWL) were measured replicatedly for three times, respectively. At the fourth day of the fifth week, all rats were sacrificed for histopathological and immunohistochemical analyses, qRT-PCR and western blot analysis.

Male Sprague Dawley (SD) rats (6 weeks, 200 ± 20 g, Shanghai Super B&K Laboratory Animal Co. Ltd., China) were used for experiments following the China legislation on the use and care of laboratory animals and approved by the Medical Norms and Ethics Committee of Zhejiang Chinese Medical University (No. SYXK (Zhejiang) 2018-0012). The rats were housed in a pathogen-free facility with a 12:12 light/dark cycle, and food and water ad libitum. To evaluate the *in vivo* efficacy of ADSCs, 30 rats were randomly divided into 3 groups: 1) normal control group (NC); 2) OA model group (Model); 3) ADSCs treated model group (ADSCs). 10 rats were in each group. OA model was generated by intra-articular injection of 50 μL 30 mg/ml monoiodoacetate (MIA) in rats (7 weeks old) once and allowed 1 week of recovery period, as described previously (Zuxiang Chen et al., 2021). After 1 week post OA modeling, rats in the NC group were intra-articularly injected with 50 μL of saline, while rats in the ADSCs group were intra-articularly treated with 50 μL of ADSCs (5 × 10^5^ cells). All treatments were performed once a week, on the first day for four consecutive weeks (Figure 1).

### Isolation of ADSCs

About 10^5^ of primary ADSCs were isolated from the inguinal adipose tissue in five rats, according to a previously reported method (Zuxiang Chen et al., 2021). Briefly, rats were anesthetized with 3% pentobarbital and their inguinal adipose tissue was harvested under aseptic conditions. The adipose tissue was carefully washed three times, sliced into pieces, and further digested with 0.2% collagenase type IV for 45 min at 37°C. The obtained cells were resuspended and filtered through a 100 μm cell strainer, and then cultured in α-MEM supplemented with 10% FBS at 37°C and 5% CO_2_ for 1 week. A total of 10^6^ cells were passaged with 0.25% trypsin every 3 days until passage 3 for the following experiments.

### Flow Cytometry Analysis of ADSCs

The primary ADSCs (10^6^ cells/ml) were stained with the following antibodies: fluorescein isothiocyanate (FITC) conjugated anti-CD29, phycoerythrin (PE) conjugated anti-CD34, allophycocyanin (APC) conjugated anti-CD44, and fluorescein isothiocyanate (FITC) conjugated anti-CD90. The labeled cells were analyzed *via* a multiparameter flow cytometer (BD Accuri C6, NJ, United States).

### Pain Behavior Evaluations

At the third day of the fifth week of the experiment, the mechanical withdrawal threshold (MWT) and the thermal withdrawal latency (TWL) were measured thrice in all animals using Electronic tenderness tester (Huaibei Zhenghua, China) and Plantar Test apparatus (UgoBasile, Italy) replicatedly for three times, respectively. Briefly, rats were individually placed into the mechanical and thermal testing chambers for 30 min without any disturbance for acclimatization. For the assessment of MWT, Von Frey filaments were pressed perpendicularly against the mid-plantar surface of the hind paws of each rat for at least three times and held for 2 s. A positive response for each test was defined as the sharp withdrawal of the hind paw, and the maximal bending forces (g) of the filaments were recorded. For the assessment of TWL, a focused beam of radiant heat (up to 35°C) was positioned beneath the plantar surface of the hind paws for at least three times and held for maximal 20 s. Withdrawal latency (s) was defined as the time from the start of the heat stimulation to the sharp withdrawal of the hind paw.

### Histopathological and Immunohistochemical Analyses

All rats were sacrificed and the joints of each rat were harvested after pain behavior evaluations on the fourth day in the fifth week of the total experimental period. The joint samples were fixed with 10% formalin for 24 h, decalcified with 10% EDTA solution for 8 weeks, and embedded in paraffin. Then, 3 μm sections of each joint sample were cut and stained with Alcian Blue Hematoxylin/Orange G (ABH) or Safranin O/Fast green (SO). The grade of OA progression was evaluated by double-blind observation, according to Mankin’s scoring systems. Immunohistochemistry assay was applied to detect the expression of matrix metallopeptidase 13 (MMP13), collagenase type II (Collagen II), and collagenase type X (Collagen X) in cartilage. The following primary antibodies against rat MMP13 (mouse anti-MMP13 monoclonal antibody, NBP2-45887, Novus), Collagen II (rabbit anti-Collagen II monoclonal antibody, ab34712, abcam), Collagen X (rabbit anti-Collagen X monoclonal antibody, ab58632, abcam), and peroxidase-conjuncted secondary antibodies (PV-9002 for MMP13, and PV-9001 for Collagen X and Collagen II) (ZSGQ-BIO, Beijing, China) were used, followed by colorimetric visualization with diaminobenzidine solution (Invitrogen, MD, United States). The immunoreactivity of MMP13, Collagen X and Collagen II was semiquantified by Image-Pro Plus 6.0 software (Media Cybernetics, Bethesda, MD, United States) under a light microscope (NIKON 80i, Tokyo, Japan). The final results were presented as the percentage of antigen-positive cells to total cells (MMP13) or antigen-positive area to total area (Collagen II and Collagen X) in the selected fields.

### Primary Chondrocytes Preparation

About 3 × 10^5^ primary chondrocytes were isolated from the articular cartilage tissue in five rats, as previously described (Yan et al., 2019). Briefly, articular cartilage tissues from rat donors were harvested and sliced into small pieces. These pieces were digested in 0.25% trypsin for 40 min at 37°C and then treated with 0.1% collagenase II for 4 h at 37°C. Isolated cells were filtered through a 70 μm cell strainer and collected as chondrocytes. IMDM medium containing 10% FBS was used to culture the chondrocytes. A total of 10^6^ cells were passaged with 0.25% trypsin every 3 days until passage 3 for the following experiments. The chondrocytes at the end of the culture were characterized by Collagen II immunofluorescence staining, as shown in Supplementary Figure S1.

### Conditioned medium preparation

Conditioned medium (CM) of ADSCs and chondrocytes were prepared for *in vitro* experiments. Briefly, ADSCs and chondrocytes were seeded at a density of 1×10^6^ cells/10 cm dish with complete medium. The medium was refreshed at cell confluency of 70% and the cells were allowed to further grow for 48 h. Then the cell culture medium was collected, centrifuged for 10 min at 1,500 rpm, and filtered through a 0.22 μm cell strainer. The ADSCs-CM and Chondrocyte-CM were stored at −80°C for further use.

### Cell Viability Assay

Chondrocytes were divided into four groups as follows: Chondrocyte-CM group, IL-1β group, ADSCs-CM group, and ADSCs-CM + IL-1β group. To minimize the cell culture medium differences between each group, rat chondrocytes in Chondrocyte-CM group and IL-1β group were treated with Chondrocyte-CM. To establish an *in vitro* cellular model of inflammatory articular cartilage tissue, rat chondrocytes in IL-1β group and ADSCs-CM + IL-1β group were pre-treated with IL-1β (10 ng/ml) for 24 h. To evaluate the potential effects of ADSCs-CM, rat chondrocytes in ADSCs-CM group and ADSCs-CM + IL-1β group were additionally treated with ADSCs-CM for another 24 h. The cell viability of chondrocytes was determined by Cell Counting Kit-8 (CCK-8) assay at 24 and 48 h, according to the manufacturer’s manual. The cell viability was represented by the optical density (OD) value, which was measured at 450 nm with a microplate reader (Bio-Rad, CA, United States).

### Quantitative Real-Time Polymerase Chain Reaction

The relative mRNA expression of targeted genes in chondrocytes was measured using a quantitative real-time polymerase chain reaction (qRT-PCR) assay on an ABI QuantStudio™ 7 Flex Real-Time PCR System (Applied Biosystems, United States). Total RNA of chondrocytes was extracted with TRIzol reagent and quality controlled by NanoDrop2000 spectrophotometer (Thermo Scientific, United States). cDNA reverse transcription was performed by using All-in-One cDNA Synthesis SuperMix kit. The qPCR reaction system was 20 μL, comprising 10 μL SYBR^®^ Premix Ex Taq II (Tli RnaseH Plus), 0.4 μL PCR Forward Primer, 0.4 μL PCR Reverse Primer, 1 μL template cDNA and 8.2 μL ddH_2_O, and the qPCR reaction conditions included pre-incubation at 95°C for 5 min, followed by 40 cycles of denaturation at 95°C for 10 s, annealing and extension at 60°C for 30 s *β-Actin* was used as the reference gene and 2^−ΔΔCT^ method was applied to measure the relative mRNA expression (Table 1).

**TABLE 1:** Primer sequences of target genes.

Gene	Forward primer	Reverse primer
*β-Actin*	5′-CCC​GCG​AGT​ACA​ACC​TTC​T-3′	5′-CCC​GCG​AGT​ACA​ACC​TTC​T-3′
*COL2A1*	5′-CTC​AAG​TCG​CTG​AAC​AAC​CA-3′	5′-GTC​TCC​GCT​CTT​CCA​CTC​TG-3′
*COL10A1*	5′-GAT​CAT​GGA​GCT​CAC​GGA​AAA-3′	5′-CCG​TTC​GAT​TCC​GCA​TTG-3′
*MMP3*	5′-TTG​ATG​ATG​ATG​AAC​GAT​GG-3′	5′-CCT​TCT​TAC​CTC​ACT​TCC​TAT-3′
*MMP13*	5′-CTA​TGG​TCC​AGG​AGA​TGA​AGA​C-3′	5′-GTG​CAG​ACG​CCA​GAA​GAA​TCT-3′
*Aggrecan*	5′-GCA​GAC​ATT​GAT​GAG​TGC​CTC-3′	5′-CTC​ACA​CAG​GTC​CCC​TCT​GT-3′
*IL-6*	5′-CTC​TCC​GCA​AGT​AAG​TGA​A-3′	5′-GGT​ATC​CTC​TGT​GAA​GTC​TC-3′
*ADAMTS5*	5′-TGG​AGT​GTG​TGG​AGG​GGA​TA-3′	5′-CGG​ACT​TTT​ATG​TGG​GTT​GC-3′
*ADAMTS9*	5′-TAC​AGG​CAA​AGG​CTG​GTC​TC-3′	5′-CTC​AGG​TAG​CAG​GGA​TGG​AC-3′
*SOX6*	5′-CAG​GAG​ATG​CGA​CAG​TTC​TTC-3′	5′-TCT​GAG​GTG​ATG​GTG​TGG​TC-3′
*SOX9*	5′-CAT​CAA​GAC​GGA​GCA​ACT​GA-3′	5′-TGT​AGT​GCG​GAA​GGT​TGA​AG-3′

### Western Blot Analysis

Total protein of chondrocytes was extracted with lysis buffer (50 mM Tris-HCl, pH 7.4, 150 mM NaCl, 1 mM EDTA, 1% Triton and 0.1% SDS) containing phosphatase and proteinase inhibitor cocktail (Bimake, Houston, TX, United States). 10 µL total protein lysate was loaded into each gel lane. The targeted protein was separated by 6–12% sodium dodecyl sulfate polyacrylamide gel electrophoresis (SDS-PAGE) and transferred to a nitrocellulose membrane (Sartorius Stedim, Göttingen, Germany). The membrane was blocked with 5% nonfat milk in Tris-buffered saline tween (TBST) at 4°C for 2 h, followed by overnight incubation at 4°C with the following primary antibodies against β-Actin (A3854, Sigma), MMP13, Collagen II, Collagen X, IL-6 (21865-1-AP, Proteintech) and SOX9 (ab185230, abcam). Each protein was detected using X-ray film (Kodak, Tokyo, Japan) and visualized using Western Lightning^®^ Plus ECL (Perkin Elmer, Inc., Waltham, MA, United States).

### Clinical Retrospective Study

This study was approved by the Ethics Committee of the First Affiliated Hospital of Zhejiang Chinese Medical University (No. ChiCTR-OCN-15006356). The data of 21 patients who have been treated with articular injection of autologous ADSCs in Zhejiang Provincial Hospital of Chinese Medicine were collected. Informed consent has been obtained from all patients. About 50 ml of adipose tissue was harvested from each patient. ADSCs were cultured in StemPro MSC SFM (A1033201, ThermoFisher) at 37°C and 5% CO_2_, and were harvested within five passages without cryopreservation. Saline was used as vehicle for injection and 1.5 × 10^7^ ADSCs were injected into the articular. For clinical evaluation of the ADSCs outcomes, visual analog scale (VAS) and Western Ontario and McMaster Universities Osteoarthritis Index (WOMAC) were assessed one week before injection (baseline) and 2 weeks after injection. Also, magnetic resonance imaging (MRI) examination was performed at the baseline time and the follow-up (7–24 months). The inclusion criteria were as follows: 1) aged 30–80 years; 2) confirmed OA diagnosis by radiological and clinical evaluations; 3) grade 2 or above according to Kellgren-Lawrence criteria and pain intensity of grade 3 or above by VAS; and 4) not responding to at least two non-surgical treatments. The exclusion criteria were as follows: 1) body mass index (BMI) ≥35; 2) patients who received additional knee joint operation or intra-articular injection of any drug during the follow-up period; 3) patients with other types of arthritis, except OA; 4) patients with severe medical problems; 5) allergic to any substance used in the treatment; and 6) pregnant or breast-feeding.

### Statistical Analysis

SPSS13.0 software (SPSS, IL, United States) was used for data analysis. Data were presented as mean ± standard deviation (SD). The Student’s t-test or One-way analysis of variance (ANOVA) was applied to evaluate the statistically significant difference between groups. A value of *p* < 0.05 was regarded as statistically significant.

## Results

### Characterization of ADSCs

As shown in Supplementary Figure S2, flow cytometry analysis showed that cultured ADSCs had high expressions of CD29, CD44 and CD90 (all > 95%) and low expression of CD34 (<0.3%), which was consistent with previous studies (Wang et al., 2018; Zuxiang Chen et al., 2021).

### Therapeutic Effects of ADSCs on Osteoarthritic Rats

**FIGURE 2:**
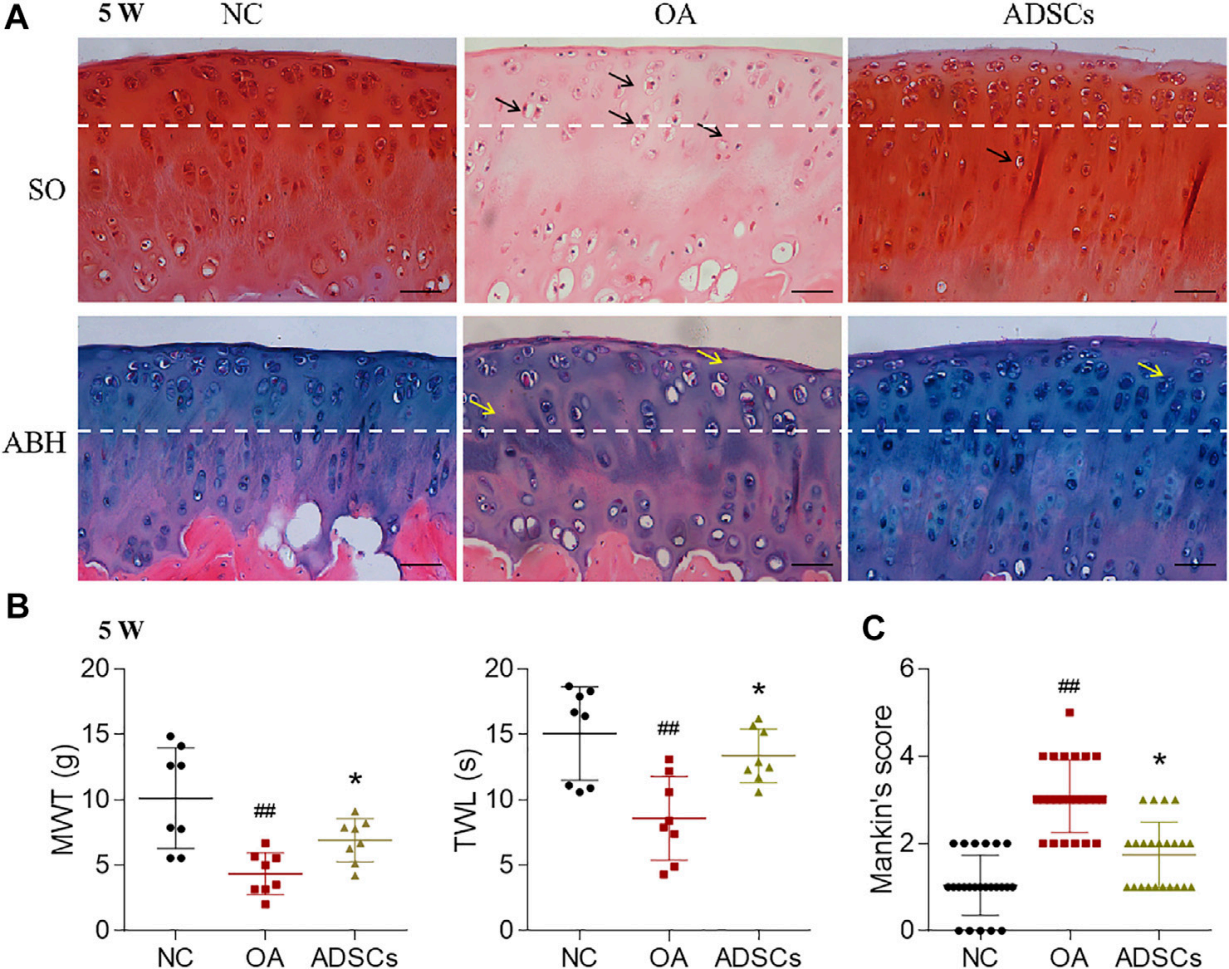
Effects of ADSCs on MIA-induced rat OA model. **(A)** Safranin O/Fast green (SO) staining and alcian blue hematoxylin (ABH) staining with black arrows (chondrocyte loss, collagen disruption) and yellow arrows (chondrocyte loss, matrix disorganization). The tissue was harvested at the fourth week after OA induction, which was the fifth week of the total experiment (5 W). The white dash line indicates the border between cartilage and subchondral bone. Scale bars = 50 μm. **(B)** Measurement of MWT and TWL of rats. *N* = 8. **(C)** Mankin’s scores for histopathological observation. Values were presented as mean ± SD. N = 24. ##*p* < 0.01 versus NC group; *p < 0.05 versus model group.

**FIGURE 3:**
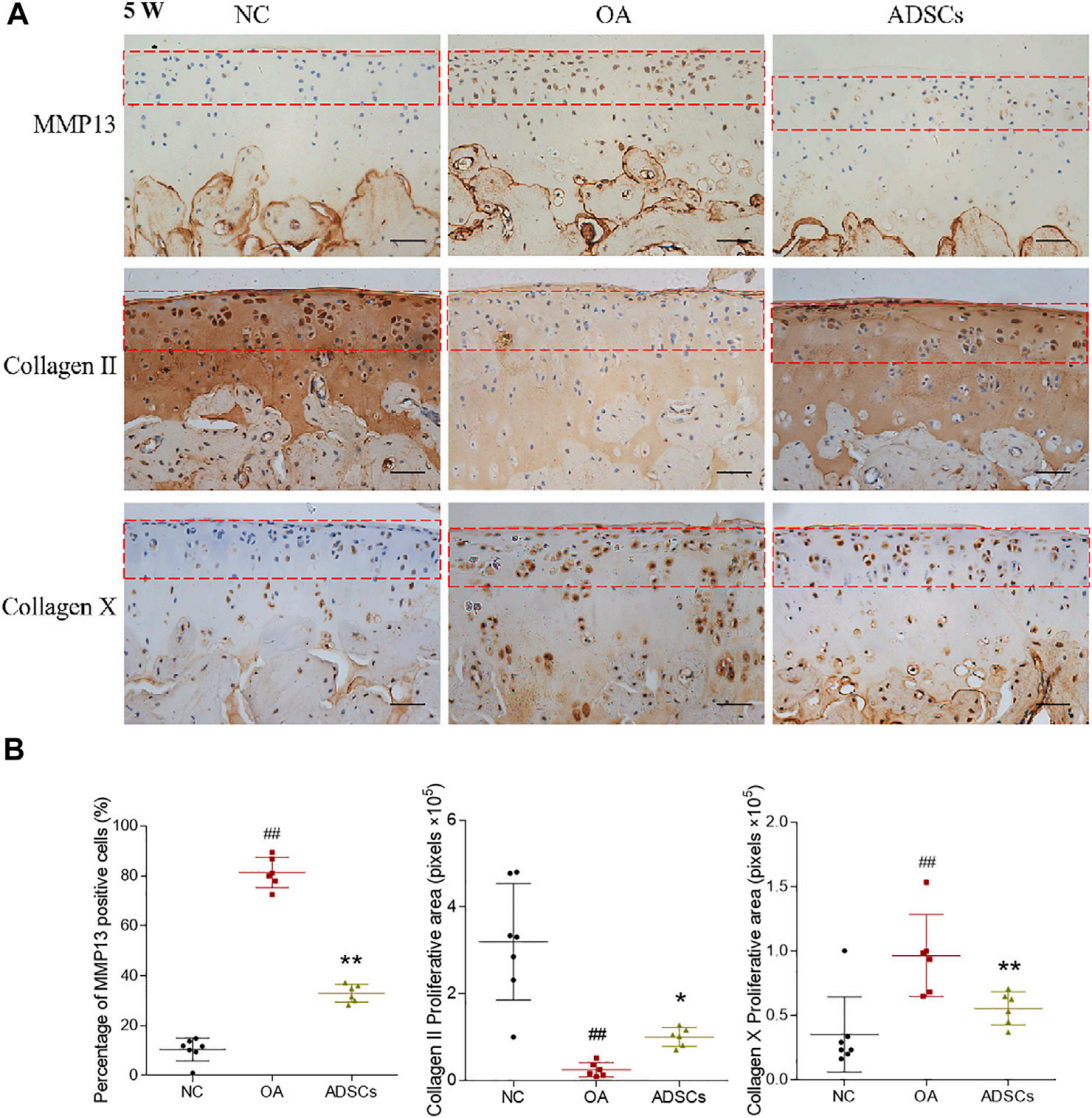
Effects of ADSCs on expressions of Collagen II, Collagen X and MMP13 on rat cartilage. **(A)** Representative immunohistochemical staining of Collagen II, Collagen X and MMP13 on cartilage at the fifth week of the experiment (5 W). Red dash box indicates region of interest where the MMP13, Collagen II and Collagen X positive cells were counted. Scale bars = 50 μm. **(B)** Positive cell percentages of MMP13 and quantitative measurement of positive area percentage of Collagen II and Collagen X. Values were presented as mean ± SD. *N* = 6. ##*p* < 0.01 versus NC group; *p < 0.05 and **p < 0.01 versus model group.

To evaluate the therapeutic effects of ADSCs on OA, a rat OA model was constructed by articular injection of MIA. After modeling, histopathological staining showed apparent cartilage degeneration reflected by chondrocyte and glycosaminoglycan loss and collagen matrix disorganization, with significantly increased Mankin’s score (*p* < 0.01 vs. NC) (Figures 2A,C). Pain behavior assessments showed abnormal mechanical allodynia and thermal hyperalgesia, reflected by the significantly decreased MWT and TWL, respectively (each *p* < 0.01 vs. NC) (Figure 2B). By contrast, intra-articular injection of ADSCs significantly increased the number of chondrocytes, improved the content of glycosaminoglycan and structural integrity of articular cartilage, with significantly reduced Mankin’s score in the ADSCs group (*p* < 0.05 vs. model) (Figures 2A,C). The ADSCs administration also restored the mechanical allodynia and thermal hyperalgesia, indicated by the significantly increased MWT and TWL (each *p* < 0.05 vs. model) (Figure 2B). Moreover, immunohistochemical results showed that MMP13 (catabolic marker), Collagen II (component of cartilage matrix), and Collagen X (hypertrophic marker) were significantly dysregulated in the model group (each *p* < 0.01 vs. NC), while the expressions of those markers were significantly reversed (each *p* < 0.01 or *p* < 0.05 vs. model) after ADSCs treatment (Figures 3A,B). Taken together, the above results suggested that a MIA-induced OA rat model was successfully established, and that ADSCs did have some functional improvement on these osteoarthritic rats.

### ADSCs Reversed the Abnormal Gene Expression of Chondrocytes From Osteoarthritic Rats

**FIGURE 4:**
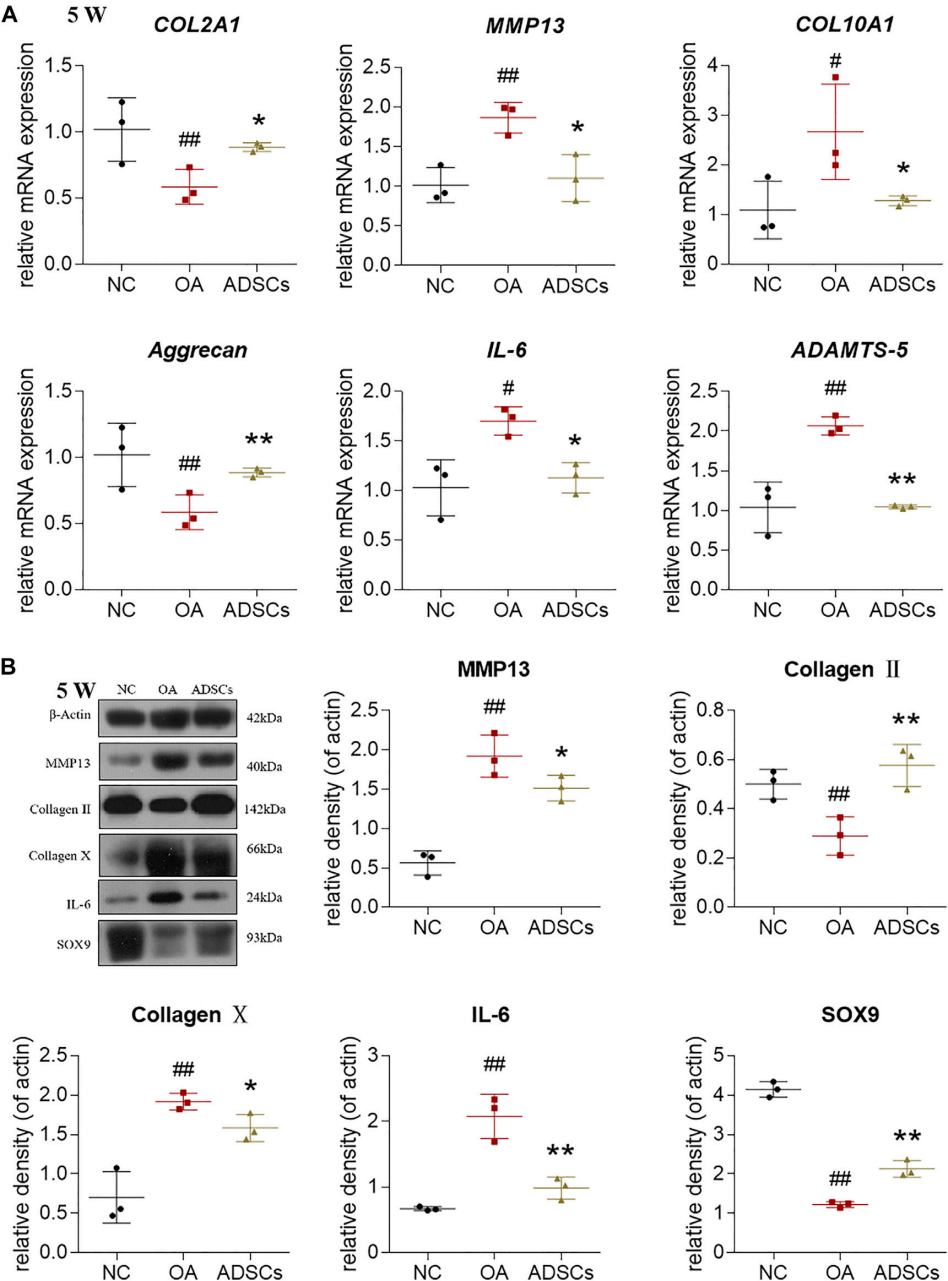
Effects of ADSCs on chondrocytes in rat cartilage. **(A)** qRT-PCR analysis of mRNA expressions of chondrocytes treated with ADSCs on rat cartilage; **(B)** Expressions of target proteins in chondrocytes treated with ADSCs on rat cartilage. Data were collected at the fifth week of the experiment (5 W). Values were presented as mean ± SD. *N* = 3. #*p* < 0.05 and ##*p* < 0.01 versus NC group; *p < 0.05 and **p < 0.01 versus model group.

To investigate the regulative effects of ADSCs on molecular levels, we used qRT-PCR and western blot to measure the gene expressions of chondrocytes from osteoarthritic rats. The qRT-PCR results showed that OA modeling significantly increased *MMP13*, *COL10A1*, *IL-6*, *ADAMTS5* mRNA expression and decreased *COL2A1*, *Aggrecan* mRNA expression in rat chondrocytes (each *p* < 0.01 or *p* < 0.05 vs. NC) (Figure 4A). These dysregulated gene expressions were significantly reversed by ADSCs treatment (each *p* < 0.01 or *p* < 0.05 vs. model) (Figure 4A). Consistent with our qRT-PCR results, western blot indicated that ADSCs significantly reversed the expression of MMP13, Collagen II, Collagen X, IL-6 and SOX9 protein in chondrocytes from osteoarthritic rats (each *p* < 0.01 or *p* < 0.05 vs. model) (Figure 4B). Thus, the above results indicated that ADSCs could effectively reverse the abnormal gene expression changes in osteoarthritic rat chondrocytes.

### Protective Effects of ADSCs-CM on IL-1β-Treated Chondrocytes

**FIGURE 5:**
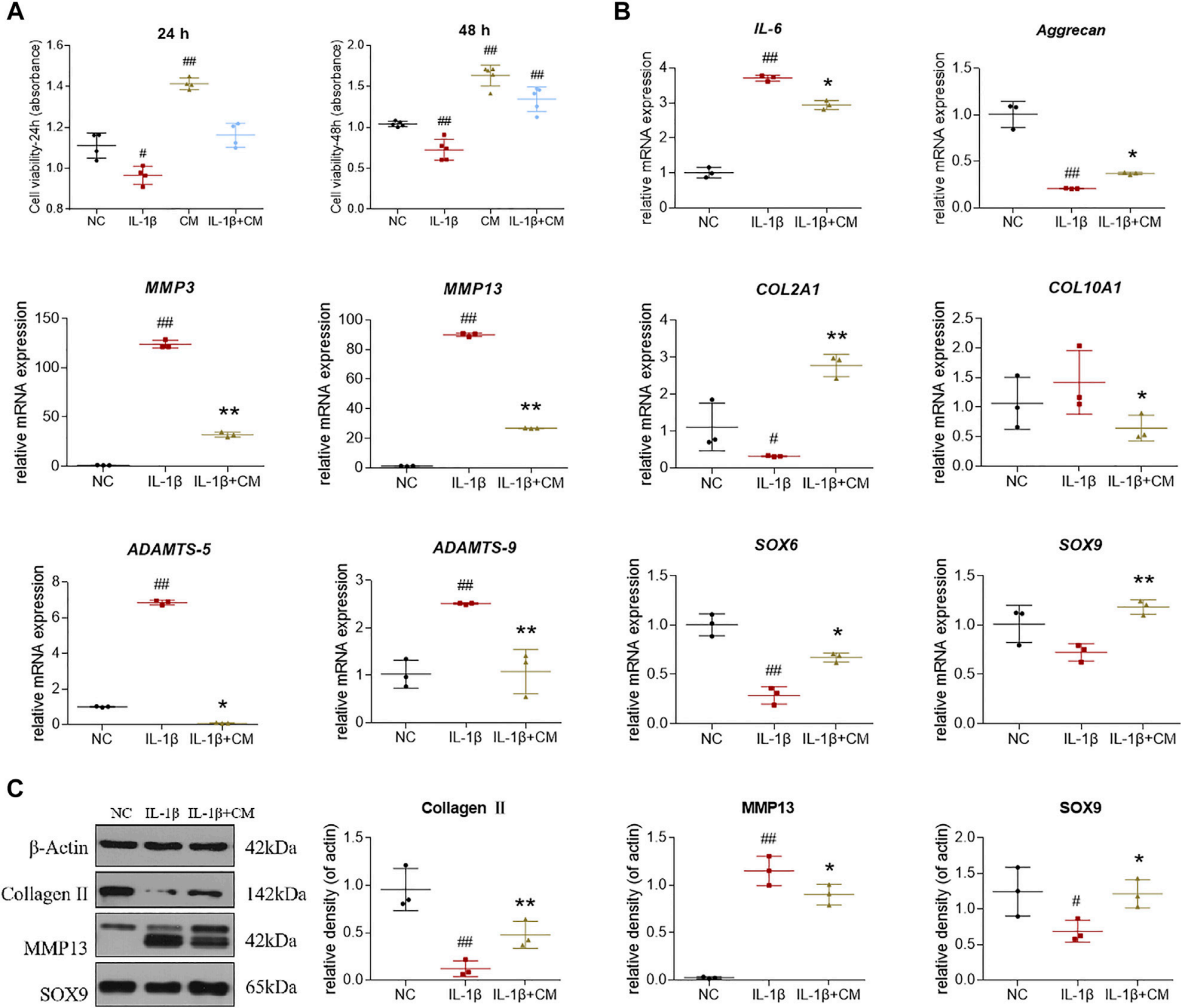
Effects of ADSCs-CM on cell viability and regulations of ADSCs-CM on IL-1β-treated chondrocytes. **(A)** Chondrocyte viability at 24 and 48 h after ADSCs-CM treatment; **(B)** qPCR analysis of mRNA expressions of chondrocytes treated with ADSCs-CM; **(C)** Expressions of target proteins in chondrocytes treated with ADSCs-CM. Values were presented as mean ± SD. *N* = 3. #*p* < 0.05 and ##*p* < 0.01 versus control group; *p < 0.05 and **p < 0.01 versus IL-1β group.

To investigate the protective effects of ADSCs-CM on cell viability, chondrocytes were pretreated with 10 ng/ml IL-1β for 24 h followed by additional treatment with ADSCs-CM for another 24 h. The results of CCK-8 assay showed that ADSCs-CM exerted obvious pro-proliferative effects on both chondrocytes and IL-1β-induced chondrocytes after 24 and 48 h treatment (Figure 5A). Then, we performed qRT-PCR to examine the regulative effects of ADSCs-CM on gene expressions in rat chondrocytes. The qRT-PCR results showed that IL-1β significantly increased *IL-6*, *MMP3*, *MMP13*, *COL10A1*, *ADAMTS5*, *ADAMTS9* mRNA expression and decreased *Aggrecan*, *COL2A1*, *SOX6*, *SOX9* mRNA expression in rat chondrocytes. These altered gene expressions were significantly reversed by ADSCs-CM after 24 h treatment (Figure 5B). Next, we examined the effects of ADSCs-CM on IL-1β-induced chondrocytes by western blot. Similarly, western blot indicated that ADSCs-CM significantly reversed the expression of Collagen II, MMP13, and SOX9 protein in IL-1β-induced chondrocytes (Figure 5C). Taken together, the above results indicated that ADSCs-CM could effectively promote the cell viability and reverse the abnormal gene expression changes induced by IL-1β in rat chondrocytes.

### Intra-Articular ADSCs Injections Relieved Symptoms in OA Patients

**FIGURE 6:**
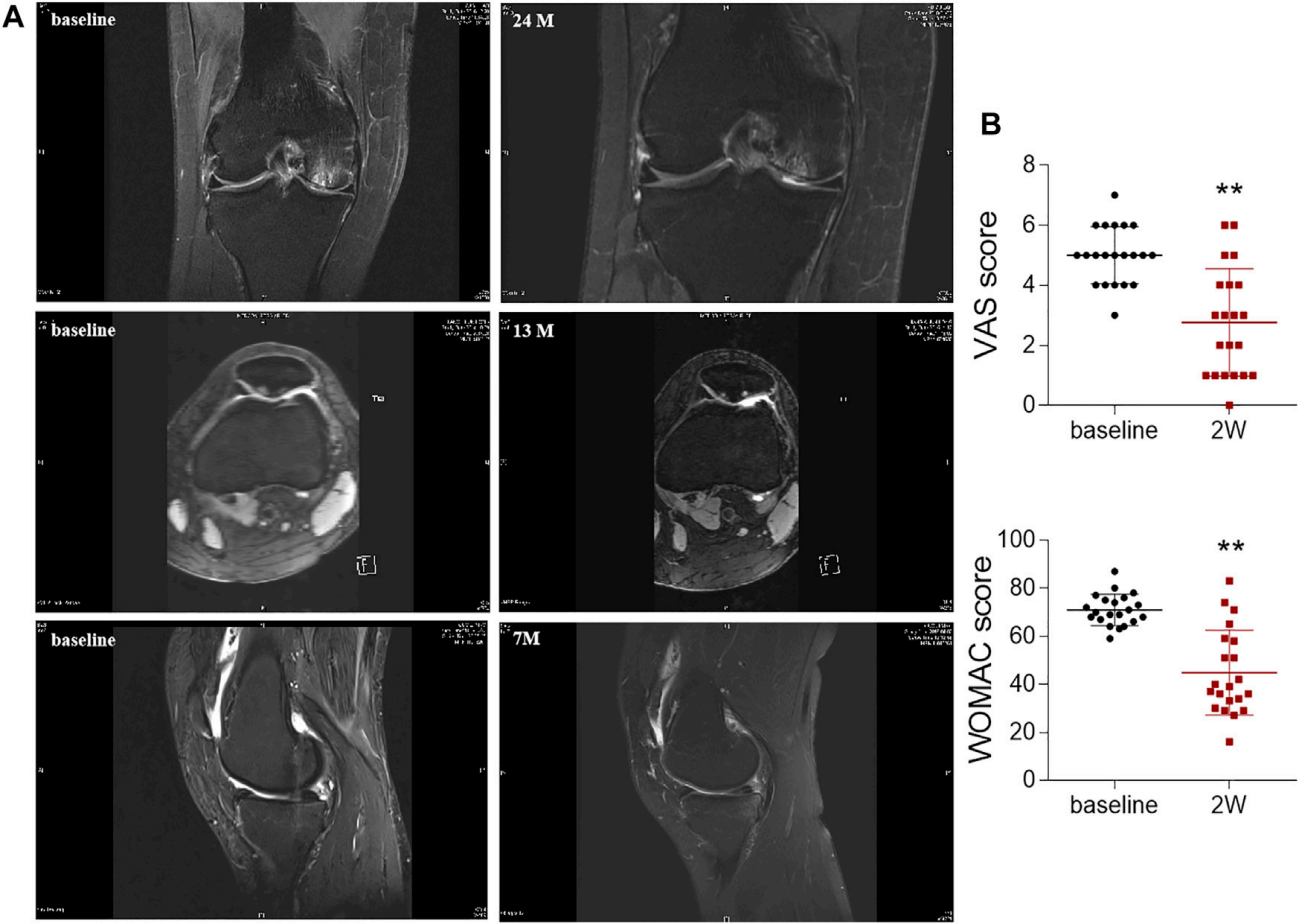
Radiological imaging analysis and OA scoring of knee joints after ADSCs treatment. **(A)** Magnetic resonance imaging evaluation of knee joint after injection. **(B)** VAS and WOMAC scores before (baseline) and after once intra-articular injection of ADSCs at week 2 (2 W). Values were presented as mean ± SD. *N* = 21. **p < 0.01 vs. Baseline group.

After validating the beneficial effects of ADSCs in rat chondrocytes, we further conducted a clinical retrospective study to evaluate the effects of intra-articular autologous ADSCs injections on articular cartilage. Of all the 21 OA patients, there was no significant difference in age, BMI, and the injection volume per joint (Table 2). All patients received an equal injection dose of 1.5 × 10^7^ ADSCs. No major injection-related adverse events were observed during the treatment and the follow-ups. For MRI review, 3 patients completed MRI evaluation at 7–24 months after ADSCs injection. MRI results revealed improved articular cartilage structure, alleviated bone marrow oedema, and reduced synovial effusion in the patient’s knee joints after ADSCs treatment (Figure 6A). Compared to the baseline measurement, the VAS score and WOMAC score measured at 2 weeks post-injection decreased by 44.8 and 36.9%, respectively, indicating a statistically significant improvement (Figure 6B).

**TABLE 2:** Clinical information of OA patients received ADSCs injection.

	Age (years)	BMI (kg/m^2^)	Left	Right	Both	A.I. vol/joint (ml)
Male (*n* = 3)	47.67 ± 24.58	27.81 ± 3.02	2	1	0	3.00 ± 1.73
Female (*n* = 18)	53.50 ± 11.74	25.15 ± 3.39	4	4	10	4.64 ± 1.76
*p* value	0.50	0.22	-	-	-	0.15

A.I., vol/joint, articular injection volume per joint.

## Discussion

Due to the variable clinical outcomes and the combined use of biological adjuncts, it is controversial whether ADSCs alone have certain efficacy against OA and how the stem cell therapy acts (Hurley et al., 2018). To answer these, the present study established an MIA-induced OA rat model and recruited OA patients to evaluate the *in vivo* anti-OA efficacy of ADSCs and applied an IL-1β-induced chondrocyte model to examine the cellular and molecular mode of action of ADSCs. The animal results demonstrated that ADSCs attenuated the progression of OA by alleviating joint pain and protecting chondrocytes and cartilage ECM from lesion (Figure 2). The *in vitro* data showed that ADSCs played chondroprotective roles by upregulating anabolic metabolism (increased expressions of Collagen II, Aggrecan, SOX6 and SOX9), downregulating catabolic metabolism (decreased expressions of ADAMTS5, ADAMTS9, MMP3 and MMP13), reducing hypertrophy (decreased expression of Collagen X), and inhibiting inflammation (decreased expression of IL-6) (Figures 3, 4). Moreover, the expression patterns of anabolic, catabolic, and hypertrophic genes on chondrocytes between ADSCs and ADSC-CM were similar, suggesting a paracrine-dependent effect of ADSCs (Figure 5). This study has the following value of science: 1) the verification of anti-OA efficacy and safety of ADSCs through the combined pre-clinical and clinical results for the first time; 2) the elucidation of regulatory roles of ADSCs towards joint pain and cartilage metabolism through an animal model of OA pain; 3) the exploration of paracrine action of ADSCs through its conditioned medium, laying the foundation for further mechanism study.

ADSCs are ideal candidate of stem cell therapy for osteoarthritis due to its paracrine excretion, chondrogenic differentiation capacity, immunological privilege, easy access, and abundant amount in adipose tissue (Alonso-Goulart et al., 2021). The chondroprotective roles of ADSCs have been proved in different OA animal models, including rat, mouse, rabbit, sheep, and goat (Tao Chen et al., 2021). For instance, an *in vivo* study reported that intra-articular injection of human ADSCs engrafted into the OA rat joints and lasted locally for over 2 months, coinciding with increased cartilage thickness (Li et al., 2016). Our *in vivo* data from OA rat model also showed statistically significant improvement in histopathological staining, pain behavior, which were in consistent with previous animal researches. Apart from the direct incorporation of ADSCs, recent studies have suggested that the paracrine effect of ADSCs played an important role in treating OA (Fazaeli et al., 2021; Zuxiang Chen et al., 2021). Our results also suggested that paracrine molecules of ADSCs exerted the anti-OA effect. ADSCs are known to secrete a variety of growth factors, cytokines, and extracellular vesicles (EVs), which have been reported to promote cell proliferation and inhibit apoptosis of chondrocytes (Liu et al., 2018). *In vitro* studies by co-culturing ADSCs and chondrocytes showed an increased secretion of many growth factors and cytokines, such as epidermal growth factor (EGF), vascular endothelial growth factor (VEGF), transforming growth factor beta (TGF-β), and bone morphogenetic protein 2 (BMP-2) of ADSCs, which in turn stimulated the synthesis of cartilage ECM (Zhong et al., 2016). ADSCs-derived EVs contain functional proteins and RNAs, such as DKK-1, versican, annexin A1, and miR-100-5p, which could induce chondrogenesis and reduce inflammation (Tofiño-Vian et al., 2018; Wu et al., 2019; Giannasi et al., 2020; Gorgun et al., 2021).

Despite the joint pain is the main symptom of OA, accompanied with degeneration of articular cartilage, most studies employing OA animal models have mainly focused on cartilage histopathology other than pain behavior (Kuroda et al., 2015; Li et al., 2016; Maumus et al., 2016; Ko et al., 2019). In this study, we not only performed the histological and immunohistochemical analyses, but also measured the MWT and TWL, which could reflect the pain response in OA models and therefore be more predictive of pain-relief efficacy of ADSCs. Our immunohistochemical results showed that some cells in the subchondral bone were positively stained for Collagen X, especially in the OA model group. It has been reported earlier that hypertrophic chondrocytes (indicated by Collagen X positive staining) could expand towards the surface of articular cartilage in osteoarthritic joints, and could be found in the subchondral bone region in young murine models (Mizoguchi et al., 1997; van der Kraan et al., 2001; Shen, 2005). To rule out these interferences, we only counted Collagen X positive cells in the cartilage region (within the red dash box, Figure 3A). In clinic, intra-articular administration of either autologous or allogeneic ADSCs has been reported to relieve pain and promote cartilage regeneration in OA patients, without any treatment-related severe adverse events (Lee et al., 2021; Maheshwer et al., 2021). For example, in a clinical trial on autologous ADSCs into the knee, the VAS and the WOMAC pain scores at 6 months post-treatment significantly decreased by 45 and 39% (each *p* < 0.01 vs. baseline) in the high-dose group (1 × 10^8^ cells), respectively (Jo et al., 2014), while not in the low-dose group (1 × 10^7^ cells). The subsequent MRI examinations found gradual regeneration of the medial femoral and tibial condyles, and thickened cartilage with isointensity over 6 months in the high-dose group (Jo et al., 2014). In another randomized double-blinded study on allogeneic ADSCs, statistically significant improvements were seen in both VAS and WOMAC pain scores in all treatment groups (*p* < 0.01), and the low-dose group (3.9 × 10^6^ cells) halted the lateral tibial cartilage loss better than the high-dose group (6.7 × 10^6^ cells) between baseline and month 12 (Kuah et al., 2018). In terms of safety, although some studies reported adverse events like transient pain or swelling of the joint after ADSCs treatment, most cases resolved spontaneously or with analgesia (Agarwal et al., 2021). Consistently, our clinical retrospective study demonstrated that articular administration of ADSCs (1.5 × 10^7^ cells) significantly reduced the pain scores (VAS and WOMAC, *p* < 0.01 vs. baseline) after 2 weeks and repaired the cartilage lesions and bone marrow oedema of knee OA patients at 7- to 24- months follow-up visits (MRI findings) (Figure 6). Compared to the routinely used intra-articular injection of sodium hyaluronate for knee OA patients in our clinic, intra-articular injection of ADSCs was superior in reducing the VAS and WOMAC pain scores. Compared to the baseline measurement, VAS and WOMAC pain scores at 2 weeks post-treatment of sodium hyaluronate decreased by 28 and 29%, respectively, which was inferior to the reduction of VAS (44.8%) and WOMAC (36.9%) scores by intra-articular injection of ADSCs in this study (Supplementary Figure S3, Supplementary Table S1). Moreover, no serious adverse event was observed during the entire treatment and follow-up periods, confirming the safety of ADSCs administration. Therefore, intra-articular injection of ADSCs is a safe and effective cell therapy for OA treatment.

Some limitations exist in this study. Inclusion of another control group of animals without OA induction but injection of ADSCs to examine the effect of ADSCs in healthy joint would have added more strength to the study. As the primary goal of this study is to evaluate the anti-OA efficacy of ADSCs, and a series of clinical studies have demonstrated the safety of intra-articular injection of ADSCs (Lee et al., 2021; Maheshwer et al., 2021), we avoided the excessive use of animals by adding a 4^th^ (additional control) group. Although we demonstrated the anti-OA efficacy of ADSCs and ADSCs-CM in the OA rat model, which bioactive component plays the major anti-OA role, and whether there are synergistic effects within the ADSCs secretome are unknown. Further, a standardized therapeutic protocol, including the isolation, culture conditions, characterization of ADSCs, injection frequency, and cell dose, is required to be set up for OA clinical treatments.

## Conclusion

This study demonstrated that ADSCs alone relieved joint pain and prevented cartilage degeneration in OA rats by restoring anabolic, catabolic, and hypertrophic genes in chondrocytes through a paracrine-based action of mode. The clinical efficacy and safety of ADSCs was further confirmed through a retrospective study. Various growth factors, cytokines, and extracellular vesicles can be released from ADSCs, but the specific roles of each component and whether they have synergistic effects remain unclear, which requires further research. Altogether, this study provides new supporting evidence of the anti-OA efficacy of ADSCs and the underlying paracrine-based actions, indicating it as a feasible stem cell-based OA therapy.

## Data Availability

The original contributions presented in the study are included in the article/Supplementary Material, further inquiries can be directed to the corresponding authors.

## Supplementary Material

Supplementary Figure S1 Characterization of chondrocytes by Collagen II immunofluorescence staining. For the immunofluorescence assay, fixed and permeabilized chondrocytes were blocked with 1% BSA in Tris-buffered saline-Tween 20 (TBST) solution for 1 h at 4°C and then incubated with Collagen II antibody overnight at 4°C. The cells were incubated with the secondary antibody for 1 h at 4°C in the dark and observed under a fluorescence microscope.

Supplementary Figure S2 Flow cytometry analysis of ADSCs.

Supplementary Figure S3 OA scoring of knee joints after sodium hyaluronate treatment. VAS and WOMAC scores before (baseline) and after once intra-articular injection of sodium hyaluronate at week 2 (2W). Values were presented as mean ± SD. *N*=21. ***P* < 0.01 vs. Baseline group. Sodium hyaluronate injection (2.5 ml/tube, certificate number: H20140533) used in our clinic was purchased from Seikagaku Corp. (Chiyoda-ku, Tokyo).

Supplementary Table S1 Clinical information of OA patients received sodium hyaluronate injection.

## Abbreviations

OA: osteoarthritis; ADSCs:adipose tissue-derived stem cells; MIA: monoiodoacetate; ACI: autologous chondrocyte implantation; MSCs: mesenchymal stem cells; ECM: extracellular matrix; CM: conditioned medium; α-MEM: Minimum essential medium-alpha modification; IMDM: Iscove’s modified Dulbecco’s medium; FBS: fetal bovine serum; PCR: polymerase chain reaction; FITC: fluorescein isothiocyanate; PE: phycoerythrin; APC: allophycocyanin; SD: Sprague Dawley; MWT: mechanical withdrawal threshold; TWL: thermal withdrawal latency; SO: Safranin O/Fast green; ABH: alcian blue hematoxylin; MMP13: matrix metallopeptidase 13; SDS-PAGE: sodium dodecyl sulfate polyacrylamide gel electrophoresis; TBST: Tris-buffered saline tween; VAS: visual analog scale; WOMAC: Western Ontario and McMaster Universities Osteoarthritis Index; MRI: magnetic resonance imaging; BMI: body mass index; EGF: epidermal growth factor; TGF-β: tumor-like growth factor-β; VEGF: vascular endothelial growth factor; BMP-2: bone morphogenetic protein 2.
