# Supplementary figures and images for: Intra-Articular Injection of Adipose-Derived Stem Cells Ameliorates Pain and Cartilage Anabolism/Catabolism in Osteoarthritis: Preclinical and Clinical Evidences
Source: Front Pharmacol. 2022 Mar 21;13:854025. doi: 10.3389/fphar.2022.854025 (PMC8978713; doi:10.3389/fphar.2022.854025)

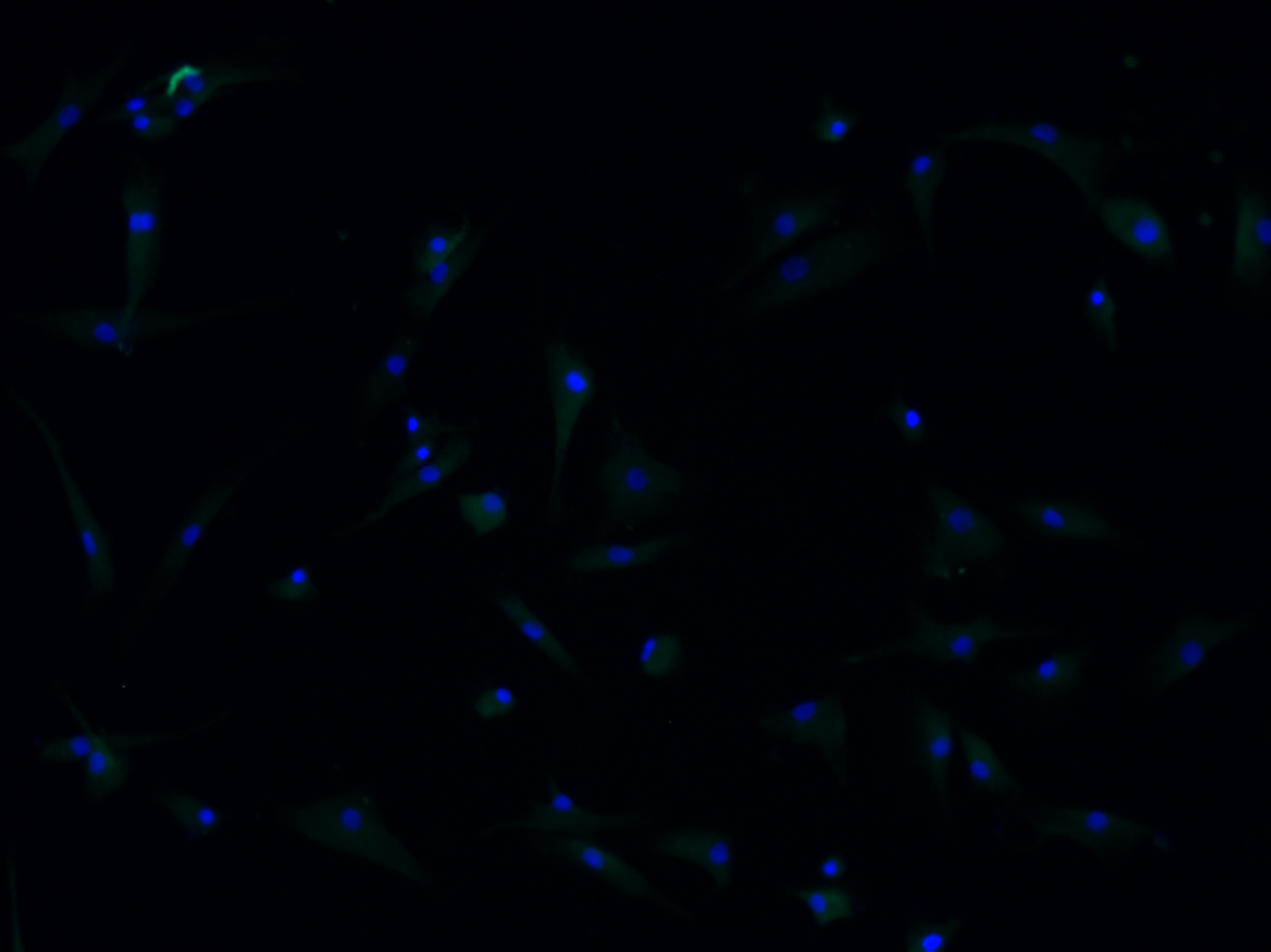

Supplement: Supplementary file 1 [file Image1.TIFF]

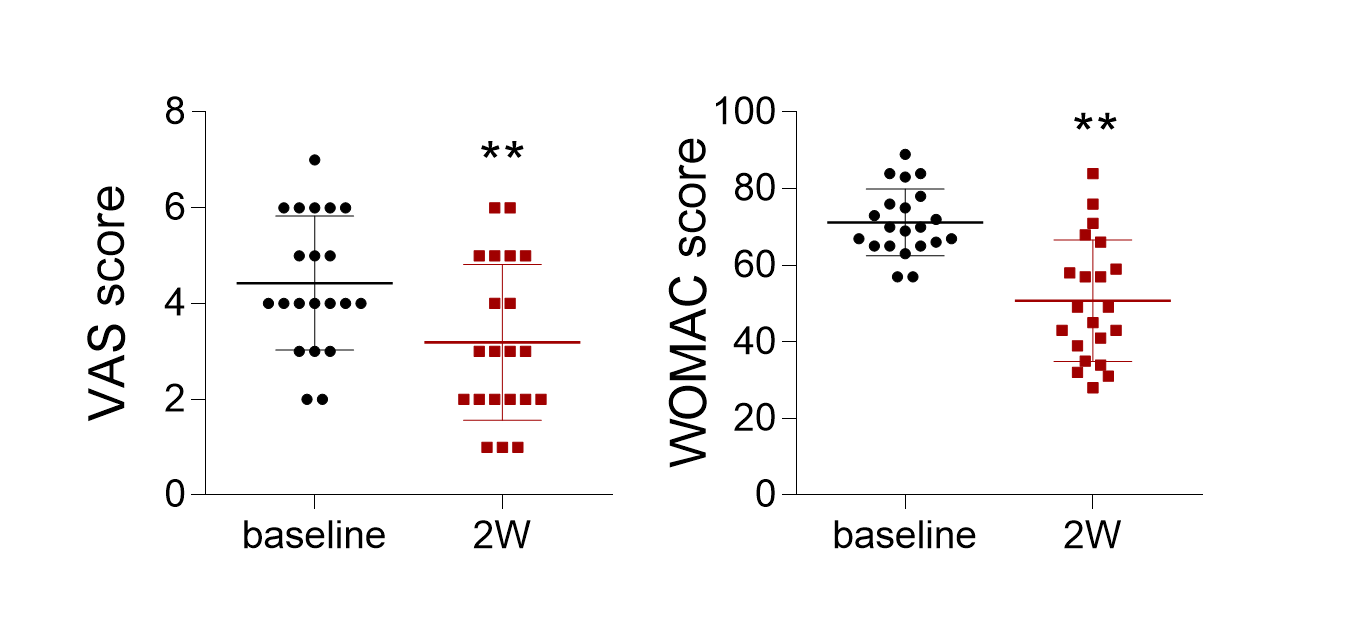

Supplement: Supplementary file 3 [file Image3.TIF]

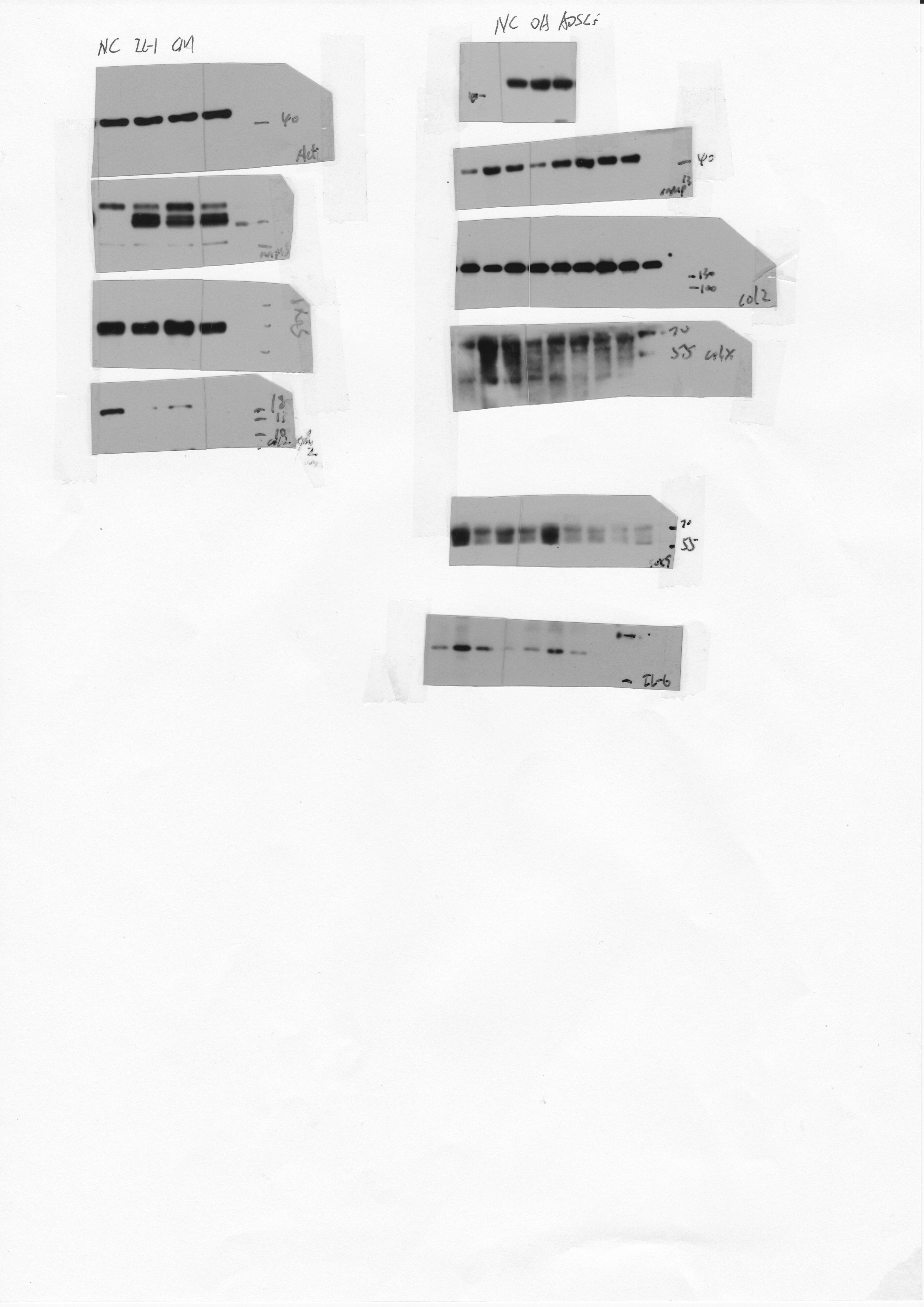

Supplement: Supplementary file 4 [file Image4.TIF]

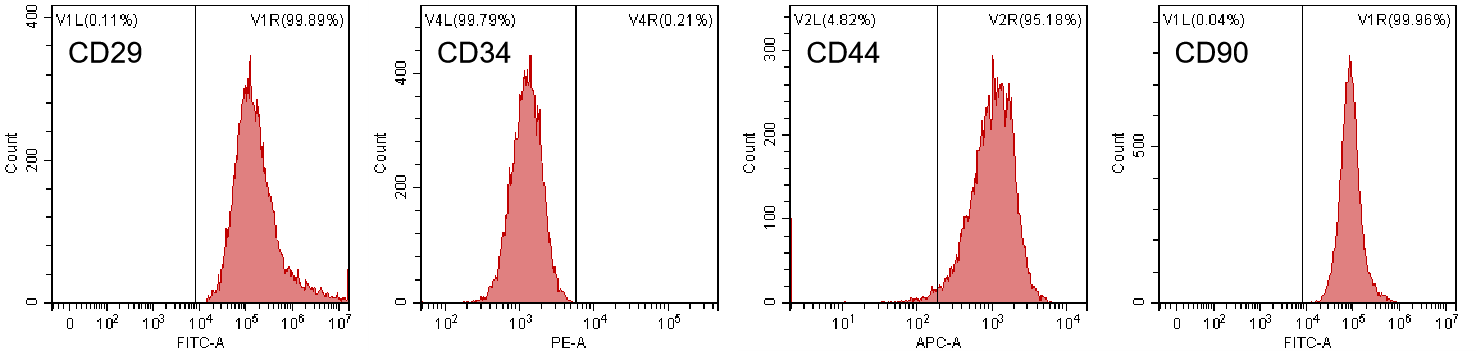

Supplement: Supplementary file 5 [file Image2.TIF]
