# Supplementary material for: Intra-Articular Injection of Adipose-Derived Stem Cells Ameliorates Pain and Cartilage Anabolism/Catabolism in Osteoarthritis: Preclinical and Clinical Evidences
Source: Front Pharmacol. 2022 Mar 21;13:854025. doi: 10.3389/fphar.2022.854025 (PMC8978713; doi:10.3389/fphar.2022.854025)
Supplement: Supplementary file 2 [file Table1.docx]

**Supplementary Table 1S.** Clinical information of OA patients received sodium hyaluronate injection.

|  | Age (years) | BMI (kg/m^2^) | Left | Right | Both | Left |
| --- | --- | --- | --- | --- | --- | --- |
| Male (n=6) | 59.67 ± 9.65 | 24.60 ± 3.23 | 0 | 5 | 1 | 0 |
| Female (n=15) | 54.87 ± 8.87 | 25.36 ± 2.99 | 8 | 5 | 2 | 8 |
| *P* value | 0.29 | 0.61 | - | - | - | - |
